# Effect of Fluence and Multi-Pass on Groove Morphology and Process Efficiency of Laser Structuring for 3D Electrodes of Lithium-Ion Batteries

**DOI:** 10.3390/ma14051283

**Published:** 2021-03-08

**Authors:** Dongkyu Park, Dongkyoung Lee

**Affiliations:** 1 Department of Future Convergence Engineering, Graduate School, Kongju National University, Cheonan 1223-24, Korea; dongkyu23@smail.kongju.ac.kr; 2 Department of Mechanical and Automotive Engineering, Kongju National University, Cheonan 1223-24, Korea

**Keywords:** lithium-ion battery, three-dimensional electrode, laser structuring, nanosecond laser, groove morphology, process efficiency

## Abstract

Lithium-ion batteries (LIBs) are widely used as energy storage systems. With the growing interest in electric vehicles, battery performance related to traveling distance has become more important. Therefore, there are various studies going on to achieve high-power and high-energy batteries. Laser structuring of electrodes involves a groove being produced on electrodes by a laser. This technique was used to show that battery performance can be enhanced due to improving Li-ion diffusion. However, there is a lack of studies about the morphological variation of grooves and process efficiency in laser parameters in the laser structuring of electrodes. In this study, the LiFePO_4_ cathode is structured by a nanosecond laser to analyze the morphological variation of grooves and process efficiency depending on laser fluence and the number of passes. First, the various morphologies of grooves are formed by a combination of fluences and the number of passes. At a fluence of 0.86 J/cm^2^ and three passes, the maximum aspect ratio of 1.58 is achieved and the surface area of structured electrodes is greater than that of unstructured electrodes. Secondly, three ablation phenomena observed after laser structuring are classified according to laser parameters through SEM images and EDX analysis. Finally, we analyze the amount of active material removal and process efficiency during laser structuring. In conclusion, applying low fluence and multi-pass is assumed to be advantageous for laser structuring of electrodes.

## 1. Introduction

Currently, rechargeable batteries such as lead–acid, nickel metal, and lithium-ion batteries (LIBs) are an essential part of smartphones, tablets, laptops, power tools, and electric vehicles. Among them, LIBs are mainly applied in various electric devices because of their relatively high energy density, high power density, cycle stability, and low memory effect [1,2]. In addition, the LIB market size is gradually expanding as wireless products are popular due to convenience, and demand for electric vehicles increases due to environmental concerns. However, there are some issues in LIBs, such as stability, energy density, and power density. In particular, electric vehicles still have a relatively shorter traveling distance and slower battery charge time than internal combustion vehicles. Thus, improving the energy density and power density of LIBs is essential to increase the performance of electric vehicles.

Several studies have been conducted to improve battery performance. Junke et al. [3] fabricated nitrogen-doped coated LiFePO_4_ by pyrolyzation treatment with egg and reported that the nitrogen-doped carbon layer effectively promoted electron and Li-ion transportation. Zelang et al. [4] fabricated an Al_2_O_3_-coated LiCoO_2_ cathode by the sol–gel method. The Al_2_O_3_ inhibited material decomposition and cobalt diffusion of the LiCoO_2_ cathode so that the energy density and cycle life of the LiCoO_2_ cathode were improved. As mentioned above, many researchers are making great efforts to improve the properties of the active material.

Meanwhile, the solution of the electrode structure, as well as the improvement of active material, is needed for high-energy and high-power of density LIBs. The energy density in LIBs increases as the amount of active material, which is the major electrochemical reaction, increases. However, when the amount of active material increases, the electrodes will be thicker, or porosity will decrease. As a result, the Li-ion diffusion distance is longer, and the wettability of electrodes is poor. These phenomena lead to reduced power density in LIBs. In short, energy density and power density have a trade-off relationship [5].

A three-dimensional (3D) electrode has been proposed to effectively overcome the issues related to battery performance, such as high interelectrode ohmic resistances resulting from longer diffusion distance, and high volume change due to lithium-ion insertion. This is because the 3D electrodes provide an additional Li-ion diffusion path and empty space in comparison to conventional 2D electrodes. The additional paths improve the Li-ion diffusion and wettability of electrodes, and the empty space on electrodes relieves mechanical stress by the volume changes [6,7,8].

Recently, laser processing has been partially applied in battery manufacturing, such as electrode cutting and battery tab and case welding, because it is possible to obtain a precise and fast process with high energy density [9,10,11,12,13,14,15,16,17,18]. In addition, laser processing has been reported as a technology that can effectively manufacture 3D electrodes. Many researchers are also working to introduce laser structuring of electrodes into the battery manufacturing process. Park et al. [19] produced grooves by a laser on LiNi_0.5_Mn_0.3_Co_0.2_O_2_ cathodes with various thicknesses (100~210 μm) and porosities (26~50%). The authors reported that the Li-ion diffusion and polarization of LiNi_0.5_Mn_0.3_Co_0.2_O_2_ cathodes improved through laser structuring, so the rate capability of cell was enhanced. Mangang et al. [20] compared the aspect ratio of the groove formed on LiFePO_4_ cathodes according to pulse duration (350 fs, 7 ps, 8 ns, 200 ns). The authors obtained the highest aspect ratio using a shorter pulse and the power density of structured LiFePO_4_ cathodes with 350 fs had the largest rate capacity improvement. Previous research mainly used a femtosecond laser, which requires high initial investment. In addition, there is a lack of analysis of various laser parameters on groove morphology, the amount of active material removal, and process efficiency.

In this paper, we fabricate 3D electrodes using a nanosecond laser, which requires relatively a low initial investment. The groove morphology and process efficiency are investigated depending on fluence and the number of passes during the laser structuring of 3D electrodes. First, the grooves are produced on LiFePO_4_ cathodes by a laser with various fluences and the numbers of passes. Next, the grooves on the laser-structured electrodes are examined in terms of morphology and chemical composition changes in the grooves by a scanning electron microscope (SEM) and energy-dispersive X-ray spectrometer (EDX). Finally, the process efficiency is analyzed by expressing the amount of active material removal and material removal rate as a function of fluence and the number of passes.

## 2. Experimental Setup

### 2.1. Design of Electrodes

**Figure 1:**
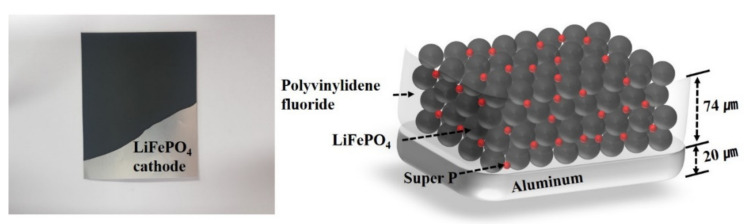
The schematic diagram of LiFePO_4_ cathode.

Figure 1 and Table 1 show the schematic and detailed information of the LiFePO_4_ cathode used in experiments, respectively. The slurry for LiFePO_4_ cathodes consists of 80 wt% LiFePO_4_, 10 wt% super P, and 10 wt% polyvinylidene fluoride. The slurry is coated on the aluminum foil, which has a 20 μm thickness. The slurry-coated aluminum foil is dried in an oven at 120 °C for 2 h. After the first drying, the electrodes are compressed by approximately 20%. Next, the second drying of electrodes is carried out at 120 °C for 24 h. The LiFePO_4_ cathode has a thickness of 94 μm (active material of 74 μm, current collector of 20 μm). The electrode-manufacturing process is carried out in a dry room with a dew point of 40 °C and temperature of 20 °C.

### 2.2. Laser Processing

**Figure 2:**
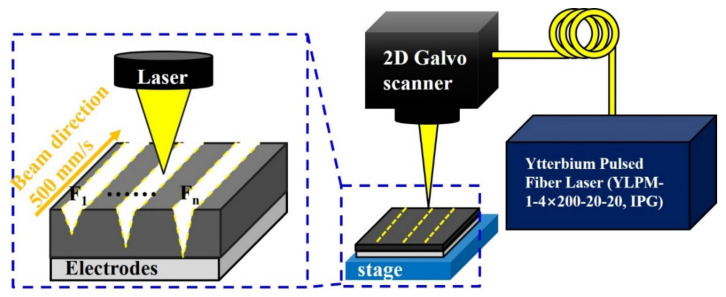
A laser processing system for the structuring of 3D electrodes.

Figure 2 shows the schematic of the system for the laser structuring of electrodes. The laser parameter used in experiments is summarized in Table 2. The ytterbium pulsed fiber laser (YLPM-1-4×200-20-20, IPG photonics, Southbridge, MA, USA) used in the structuring of the electrode has a wavelength of 1064 nm. The laser can control various parameters such as fluence, pulse duration, repetition rate, scanning speed, and the number of passes. However, only the fluence and the number of passes are controlled in this experiment. Accordingly, the pulse duration, repetition rate, and scanning speed are fixed at 4 ns, 500 kHz, and 500 mm/s, respectively. The range of the fluence is 0.28 J/cm^2^ to 5.38 J/cm^2^, and the number of passes is one pass to four passes. Pulse overlap is 96.67% and can be calculated by Equation (1).

(1) $$\mathrm{Pulse}\;\mathrm{overlap}=\;\left(1-\frac{\mathrm{Scanning}\;\mathrm{speed}\;\left[\mathrm{mm}/s\right]}{\mathrm{Repetition}\;\mathrm{rate}\;\left[\mathrm{kHz}\right]\times \mathrm{Spot}\;\mathrm{size}\left[\mu m\right]}\right)\times 100\%$$

### 2.3. Measurements

**Figure 3:**
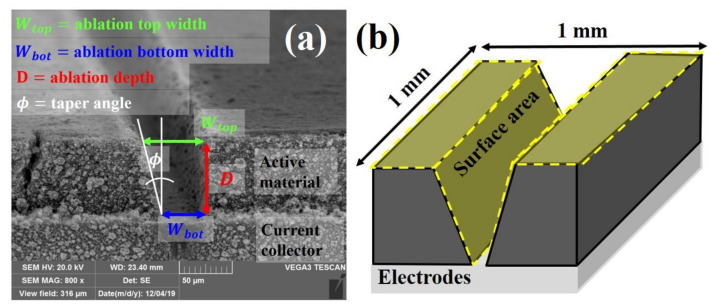
The definition of (**a**) ablation width, ablation depth, taper angle, and (**b**) surface area.

Figure 3a is obtained by SEM (TESCAN VEGA3, Brno, Czech Republic) after laser structuring. The ablation top width $\left(w_{top}\right)$, ablation bottom width $\left(w_{bot}\right)$, ablation depth $\left(D\right)$, and taper angle $\left(\phi \right)$ are defined in order to investigate the morphology of grooves depending on laser parameters. $w_{top}$, $w_{bot}$, D, and ϕ represent the dimensions of the groove on active material removed by a laser beam. In Figure 3b, the surface area is defined as the total area of the exposed surface within an area of 1 ${\mathrm{mm}}^{2}$ on the surface. The surface area can be calculated by Equation (2).

(2) $$\mathrm{Surface}\;\mathrm{area}\;\left[{\mathrm{mm}}^{2}\right]=1\;{\mathrm{mm}}^{2}+\left(\frac{D}{\cos\left(\phi \right)}\times 1\;\mathrm{mm}\times 2\right)-\left(w_{top}\times 1\;\mathrm{mm}\right)$$

## 3. Results and Discussion

### 3.1. Groove Morphology Analysis

**Figure 4:**
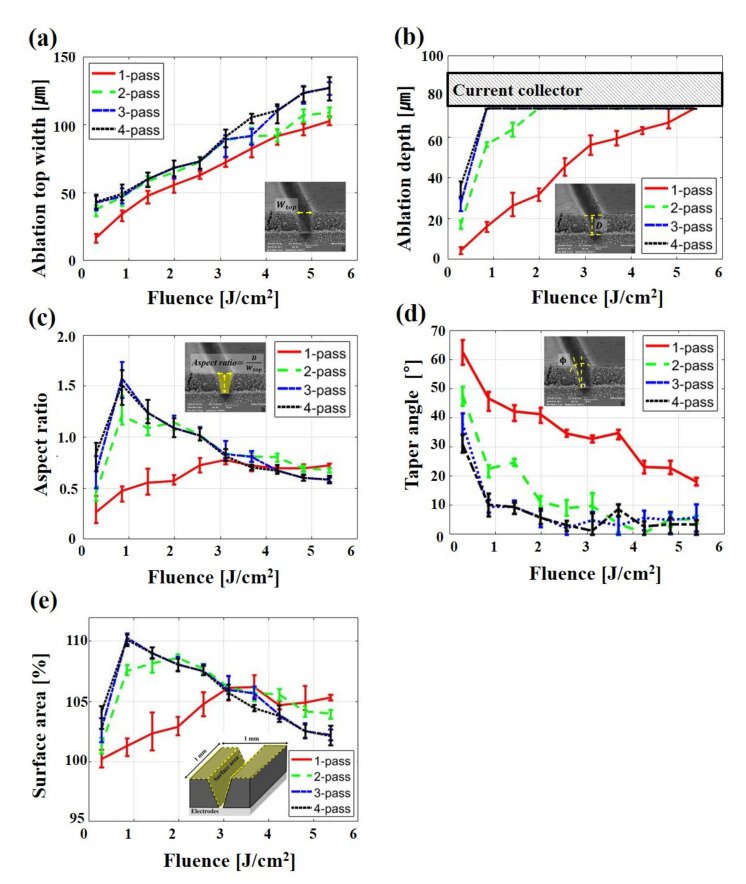
The variation of (**a**) ablation top width, (**b**) ablation depth, (**c**) aspect ratio, (**d**) taper angle, (**e**) surface area depending on fluence and the number of passes.

Figure 4 shows the variation of $w_{top}$, D, ϕ, aspect ratio, and surface area depending on fluence and the number of passes. As shown in Figure 4a, the $w_{top}$ gradually increases as fluence and the number of passes increase. With the given parameters, the $w_{top}$ is formed in the range of approximately 16.6 μm (half the size of the spot) to 127.1 μm (four times the size of the spot).

In Figure 4b, the tendency of D is similar to that of $w_{top}$. However, the D rapidly increases as the number of passes increases at the same fluence. When applying one, two, three, or four passes for laser structuring, the fluence requires 5.38 J/cm^2^, 1.98 J/cm^2^, 0.86 J/cm^2^, and 0.86 J/cm^2^ to fully remove the active material layer, respectively. In addition, molten aluminum foil is observed at two, three, and four passes. This is because the aluminum foil is affected by the extra pass after the active material is completely removed. This result is discussed in more detail in the next part.

Figure 4c shows the aspect ratio calculated by $w_{top}$ and D. In the laser structuring of electrodes, the analysis of the aspect ratio is important, because the aspect ratio of the groove in electrodes affects the power density of LIBs [20]. As the fluence increases at one pass, the aspect ratio gradually increases and achieves a maximum aspect ratio of approximately 0.78 at 3.11 $J/{\mathrm{cm}}^{2}$. Moreover, when applying multi-pass, the maximum aspect ratio is achieved 1.21 for two passes, 1.58 for three passes, and 1.51 for four passes at a fluence of 0.86 $J/{\mathrm{cm}}^{2}$. In both cases, the aspect ratio gradually decreases after achieving the maximum aspect ratio. This is because D no longer increases after the active material is completely removed, while $w_{top}$ continues to widen.

In Figure 4d, ϕ is reduced as the fluence and the number of passes increase. When using one pass, ϕ decreases to 19° in a given fluence. When using multi-pass, ϕ is reduced to almost 0°. The current collector and active material are vertical at this time. As shown in Figure 4e, the tendency of the surface area is similar to that of the aspect ratio (Figure 4c). The surface area of structured electrodes is approximately 10% greater than that of unstructured electrodes at three and four passes when a fluence of 0.86 $J/{\mathrm{cm}}^{2}$ is applied. This result implies that the maximum surface area of structured electrodes is achieved when the maximum aspect ratio is formed on the structured electrodes.

**Figure 5:**
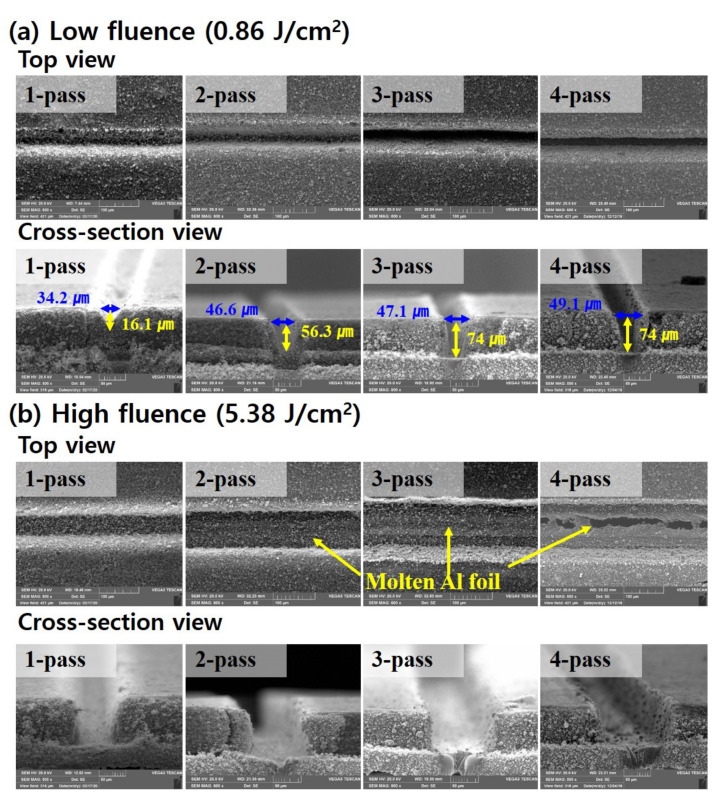
SEM images of laser-structured LiFePO_4_ cathode with (**a**) Low fluence (0.86 J/cm^2^); (**b**) high fluence (5.38 J/cm^2^).

In Figure 5, the effect of the number of passes on laser structuring of 3D electrodes is analyzed at fluences of 0.86 $J/{\mathrm{cm}}^{2}$ and 5.38 $J/{\mathrm{cm}}^{2}$ which are relatively low and high fluences, respectively. As can be seen in Figure 5a, with the fluence of 0.86 $J/{\mathrm{cm}}^{2}$, the three passes are required to completely remove active material with a thickness of 76 μm. As the number of passes increases at the fluence of 0.86 $J/{\mathrm{cm}}^{2}$, the variation of$\;w_{top}$ is 12.4 μm (from one to two passes), 0.5 μm (from two to three passes), and 2.0 μm (from three to four passes). On the other hand, the variation of D is 40.2 μm (from one to two passes), 17.7 μm (from two to three passes), and 0 μm (from three to four passes). Namely, when the number of passes increases, the D is more influenced by the laser scan than $w_{top}$ until the active material is completely removed. Therefore, it can be assumed that a groove with a higher aspect ratio can be easily achieved by low fluence and multi-pass.

In Figure 5b, the active material is completely removed by one scan with 5.38 $J/{\mathrm{cm}}^{2}$. However, the aspect ratio of the groove is relatively low except for one pass at the same laser fluence. Furthermore, the molten aluminum is observed at two and three passes. In particular, the electrodes are partially cut at four passes. These results are related to the ablation threshold of the aluminum foil. The ablation threshold means that the minimum energy per unit surface is required to induce detectable changes in materials [21]. Previous researchers reported that the ablation threshold of aluminum foil was 4.0~5.2 $J/{\mathrm{cm}}^{2}$ at a pulse duration of 4.5~5.0 ns with a wavelength of 1064 nm [22,23]. Based on the reported ablation threshold, it can be assumed that after active material is completely removed with one pass, the aluminum foil is melted by the laser beam of extra passeswhich laser fluence is higher than the ablation threshold of the aluminum foil. In the event of such damage to the aluminum foil, multi-pass should be avoided at a relatively high fluence such as 5.38 $J/{\mathrm{cm}}^{2}$ because it may reduce the battery performance due to molten aluminum contaminants [24].

### 3.2. Classification of the Ablation Region

**Figure 6:**
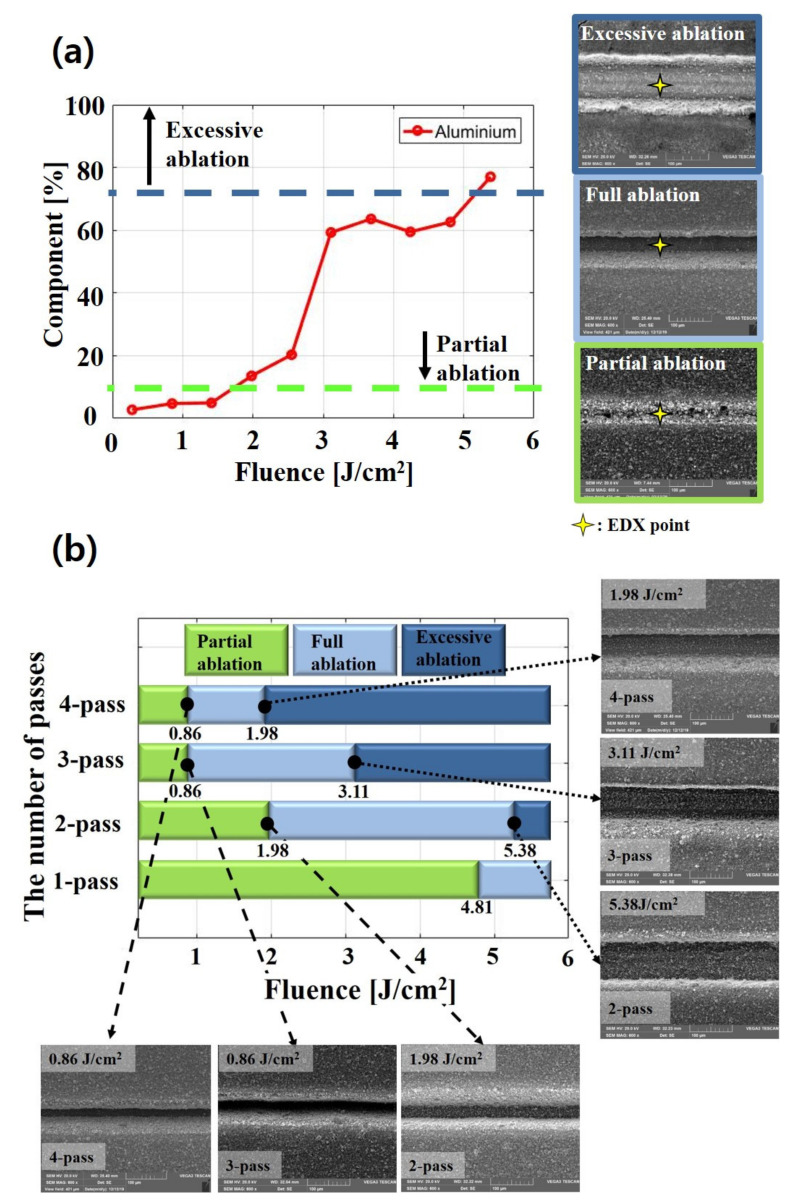
(**a**) Energy-dispersive X-ray (EDX) analysis of structured LiFePO_4_ cathode with two passes and (**b**) classification of the ablation region.

In Section 3.2, three ablation phenomena observed within experimental parameters are analyzed and classified according to fluence and the number of passes. Partial ablation is defined as when the active material layer is shallowly removed by a laser beam. Full ablation means that the active material in electrodes is completely removed by a laser beam. When the active material is completely removed and aluminum is damaged by a laser beam, this is defined as excessive ablation. The SEM images of each phenomenon are presented in Figure 6.

Figure 6a shows the variation of an aluminum component in partial ablation, full ablation, and excessive ablation. Energy-dispersive X-ray (EDX) analysis is conducted on structured electrodes with two passes. The aluminum component is detected on grooves that are formed by a laser. In the partial ablation region, the aluminum component increases gradually from 2.75% to 4.95% as the fluence increases from 0.28 $J/{\mathrm{cm}}^{2}$ to 1.41 $J/{\mathrm{cm}}^{2}$. This is because when the electrodes are irradiated with higher fluence, a deeper groove is formed. The aluminum component is detected as approximately 13.55~63.59% in a full ablation region of 1.98 $J/{\mathrm{cm}}^{2}$~4.81 $J/{\mathrm{cm}}^{2}$ and the excessive ablation region is detected in over 77.04% of the aluminum component. When the pure aluminum foil is used for EDX analysis, the aluminum component is approximately 98.7%. These results implied that LiFePO_4_ is not completely removed and residues are present after laser structuring.

Figure 6b shows three observed ablation phenomena that are classified depending on laser parameters, and the SEM images of the laser parameters at which each ablation phenomenon begins to be observed are presented. When applying one pass, partial ablation is observed below a fluence of 4.81 $J/{\mathrm{cm}}^{2}$ and full ablation occurs over a fluence of 4.81 $J/{\mathrm{cm}}^{2}$. Full ablation occurs from a fluence of 0.86 $J/{\mathrm{cm}}^{2}$ in three and four passes. In addition, excessive ablation occurs over a fluence of 3.11 $J/{\mathrm{cm}}^{2}$ for three passes and a fluence of 1.98 $J/{\mathrm{cm}}^{2}$ for four passes. As the number of passes increases, excessive ablation occurs at lower fluences. It could be assumed that the ablation threshold of aluminum foil decreases due to heat accumulation [25,26].

### 3.3. Amount of Material Removal (AMR) and Material Removal Rate (MRR)

**Figure 7:**
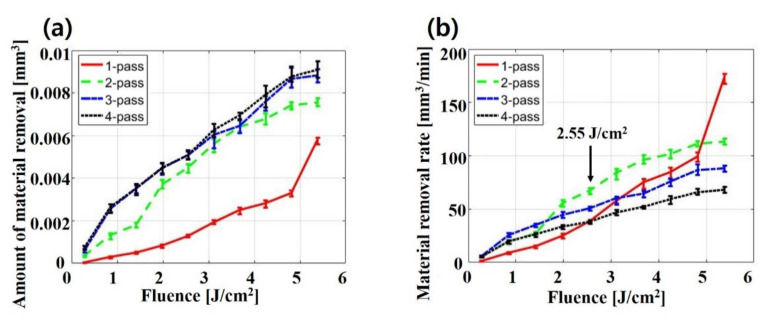
(**a**) Amount of material removal (AMR) and (**b**) material removal rate (MRR) depending on laser parameters.

Figure 7 shows the correlation between laser parameters and the amount of material removal (AMR) and the material removal rate (MRR). The AMR is calculated by Equation (3). The MRR is obtained by Equation (4) and is divided by the number of passes to analyze the MRR per pass.

(3) $$\mathrm{Amount}\;\mathrm{of}\;\mathrm{material}\;\mathrm{removal}\;\left[{\mathrm{mm}}^{3}\right]=\frac{1}{2}\left(w_{top}+w_{bot}\right)\;\times D\times 1\;\mathrm{mm}$$

(4) $$\mathrm{Material}\;\mathrm{removal}\;\mathrm{rate}\;\left[\frac{{\mathrm{mm}}^{3}}{\min}\right]=\frac{1}{2}\left(w_{top}+w_{bot}\right)\;\times D\times v\times \frac{1}{\mathrm{the}\;\mathrm{number}\;\mathrm{of}\;\mathrm{pass}}$$

As can be seen in Figure 7a, the AMR increases as higher fluences and multi-pass are applied. Namely, irradiating with a laser beam of high fluence with multiple passes not only forms a relatively lower aspect ratio, but also increases the loss of active material. It could reduce the energy density of the battery without providing any benefit from laser structuring. Therefore, applying multi-pass at low fluence in the laser structuring of electrodes with a nanosecond laser is considered as an option to minimize the loss of active material.

In Figure 7b, the MRR increases as the fluence increases at all passes. In addition, the effect of the number of passes on the MRR is significantly different depending on the fluence. When the fluence is less than 2.55 $J/{\mathrm{cm}}^{2}$, the MRR of multi-pass is higher than that of one pass. However, when the fluence is over 2.55 $J/{\mathrm{cm}}^{2}$, the MRR of one pass rapdly increases so that one pass is eventually the highest at a fluence of 5.38 $J/{\mathrm{cm}}^{2}$. These results may be because ablation efficiency per pass increases due to the heat accumulation effect until the active material is completely removed at each number of passes. On the contrary, after the active material is fully removed with multi-pass, the ablation efficiency of multi-pass decreases because of an extra pass. In addition, if the total energy is defined as fluence × the number of passes, the total energy at which the active material begins to be completely removed is 4.81 $J/{\mathrm{cm}}^{2}$ for one pass, 3.96 $J/{\mathrm{cm}}^{2}$ for two passes, 2.55 $J/{\mathrm{cm}}^{2}\;$ for three passes, and 3.40 $J/{\mathrm{cm}}^{2}\;$ for four passes. In terms of total energy, multi-pass is also more efficient to completely remove active material because less total energy is needed.

## 4. Conclusions

The laser structuring of electrodes has been proposed as one of the ways to overcome the structure issue of LIBs. In this paper, grooves are produced on LiFePO_4_ cathodes by a nanosecond laser. The effect of fluence and the number of passes on the morphology of the grooves is investigated. In addition, the three ablation phenomena, partial ablation, full ablation, and excessive ablation, are identified together with the input laser parameters. To evaluate process efficiency, the AMR and MRR are analyzed. This study is summarized as follows:

1. The groove in electrodes is formed widely and deeply as the fluence and the number of passes increase. The maximum aspect ratio of 1.58 is achieved at three passes and a fluence of 0.86 $J/{\mathrm{cm}}^{2}$, increasing the surface area by approximately 10% more than that of unstructured electrodes.
2. When increasing the number of passes with a relatively low fluence of 0.86$\;J/{\mathrm{cm}}^{2}$, it is easy to form a groove with a high aspect ratio. On the other hand, as multi-pass with a relatively high fluence of 5.38 $J/{\mathrm{cm}}^{2}$ is applied, a groove with a lower aspect ratio is formed and the current collector is damaged.
3. The ranges of the aluminum content which are detected in partial ablation, full ablation, and excessive ablation are from 2.75% to 4.95%, from 13.55% to 63.59%, and 77.04% and more, respectively. As the laser scan increases, excessive ablation occurs with relatively low fluence.
4. The higher fluence and multi-pass lead to more reduced active material during laser structuring. The MRR is significantly different according to the combination of fluence and the number of passes. Considering both the AMR and MRR at the same time, low fluence and multi-pass in the laser structuring of electrodes are assumed to be effective.

Consequently, it is assumed that applying multi-pass in low fluence will be advantageous to achieve a high aspect ratio, a relatively low amount of material removal, and high processing efficiency. As the future works of this study, battery performance tests on rate capability will be conducted.

## Figures and Tables

**Table 1:** The information of LiFePO_4_ cathode.

Classification	Value
Active material	LiFePO_4_ (80 wt%)
Conducting agent	Super P (10 wt%)
Binder	Polyvinylidene fluoride (10 wt%)
Active material thickness	90 μm → 74 μm
Current collector	Aluminum foil
Current collector thickness	20 μm

**Table 2:** Laser parameters used in experiments.

Ytterbium Pulsed Fiber Laser (YLPM-1-4×200-20-20, IPG)	Value
Fluence	0.28~5.38 J/cm^2^
Wavelength	1064 nm
Pulse duration	4 ns
Repetition rate	500 kHz
Scanning speed	500 mm/s
Pulse overlap	96.67%
The number of passes	1~4 passes

## Data Availability

Data sharing not applicable.

## References

[1] Nitta N., Wu F., Lee J.T., Yushin G. (2015). Li-ion battery materials: Present and future. Mater. Today.

[2] Zubi G., Dufo-López R., Carvalho M., Pasaoglu G. (2018). The lithium-ion battery: State of the art and future perspectives. Renew. Sustain. Energy Rev..

[3] Ou J., Yang L., Jin F., Wu S., Wang J. (2020). High performance of LiFePO4 with nitrogen-doped carbon layers for lithium ion batteries. Adv. Powder Technol..

[4] Jian Z., Wang W., Wang M., Wang Y., Auyeung N., Liu M., Feng Z. (2018). Al_2_O_3_ coated LiCoO_2_ as cathode for high-capacity and long-cycling Li-ion batteries. Chin. Chem. Lett..

[5] Zheng H., Li J., Song X., Liu G., Battaglia V.S. (2012). A comprehensive understanding of electrode thickness effects on the electrochemical performances of Li-ion battery cathodes. Electrochim. Acta.

[6] Pfleging W. (2018). A review of laser electrode processing for development and manufacturing of lithium-ion batteries. Nanophotonics.

[7] Habedank J.B., Schwab D., Kiesbauer B., Zaeh M.F. (2020). Paving the way for industrial ultrafast laser structuring of lithium-ion battery electrodes by increasing the scanning accuracy. J. Laser Appl..

[8] Zhu P., Seifert H.J., Pfleging W. (2019). The ultrafast laser ablation of Li(Ni_0.6_Mn_0.2_Co_0.2_)o_2_ electrodes with high mass loading. Appl. Sci..

[9] Lee D. (2018). Investigation of Physical Phenomena and Cutting Efficiency for Laser Cutting on Anode for Li-Ion Batteries. Appl. Sci..

[10] Jansen T., Kandula M.W., Hartwig S., Hoffmann L., Haselrieder W., Dilger K. (2019). Influence of Laser-Generated Cutting Edges on the Electrical Performance of Large Lithium-Ion Pouch Cells. Batteries.

[11] Kronthaler M., Schloegl F., Kurfer J., Wiedenmann R., Zaeh M., Reinhart G. (2012). Laser Cutting in the Production of Lithium Ion Cells. Phys. Procedia.

[12] Lutey A.H., Fortunato A., Carmignato S., Fiorini M. (2017). High speed pulsed laser cutting of LiCoO_2_ Li-ion battery electrodes. Opt. Laser Technol..

[13] Zhang Y., Li J., Yang R., Liu T., Yan Y. (2019). Analysis of kerf quality on ultrafast laser cutting of anode material for lithium-ion battery. Opt. Lasers Eng..

[14] Schmitz P., Habedank J.B., Zaeh M.F. (2018). Spike laser welding for the electrical connection of cylindrical lithium-ion batteries. J. Laser Appl..

[15] Lee D., Mazumder J. (2018). Effects of momentum transfer on sizing of current collectors for lithium-ion batteries during laser cutting. Opt. Laser Technol..

[16] Jansen T., Kandula M.W., Blass D., Hartwig S., Haselrieder W., Dilger K. (2020). Evaluation of the Separation Process for the Production of Electrode Sheets. Energy Technol..

[17] Trinh L.N., Lee D. (2020). The Characteristics of Laser Welding of a Thin Aluminum Tab and Steel Battery Case for Lithium-Ion Battery. Metals.

[18] Lee D., Patwa R., Herfurth H., Mazumder J. (2016). Parameter optimization for high speed remote laser cutting of electrodes for lithium-ion batteries. J. Laser Appl..

[19] Park J., Hyeon S., Jeong S., Kim H.J. (2019). Performance enhancement of Li-ion battery by laser structuring of thick electrode with low porosity. J. Ind. Eng. Chem..

[20] Mangang M., Seifert H., Pfleging W. (2016). Influence of laser pulse duration on the electrochemical performance of laser structured LiFePO4 composite electrodes. J. Power Sources.

[21] Luetke M., Franke V., Techel A., Himmer T., Klotzbach U., Wetzig A., Beyer E. (2011). A Comparative Study on Cutting Electrodes for Batteries with Lasers. Phys. Procedia.

[22] Porneala C., A Willis D. (2009). Time-resolved dynamics of nanosecond laser-induced phase explosion. J. Phys. D Appl. Phys..

[23] Vlǎdoiu I., Stafe M., Neguţu C., Popescu I.M. (2008). Nanopulsed ablation rate of metals dependence on the laser fluence and wavelength in atmospheric air. UPB Sci. Bull. Ser. A Appl. Math. Phys..

[24] Mohanty D., Hockaday E., Li J., Hensley D.K., Daniel C., Wood D.L. (2016). Effect of electrode manufacturing defects on electrochemical performance of lithium-ion batteries: Cognizance of the battery failure sources. J. Power Sources.

[25] Raciukaitis G., Brikas M., Gecys P., Gedvilas M. (2008). Accumulation effects in laser ablation of metals with high-repetition-rate lasers. High-Power Laser Ablation VII.

[26] Lee D. (2018). Understanding of BeCu Interaction Characteristics with a Variation of ns Laser-Pulse Duration. Materials.

